# Generation and Characterization of Mice Expressing a Conditional Allele of the Interleukin-1 Receptor Type 1

**DOI:** 10.1371/journal.pone.0150068

**Published:** 2016-03-01

**Authors:** Matthew J. Robson, Chong-Bin Zhu, Meagan A. Quinlan, David A. Botschner, Nicole L. Baganz, Kathryn M. Lindler, Jason G. Thome, William A. Hewlett, Randy D. Blakely

**Affiliations:** 1 Department of Pharmacology, Vanderbilt University School of Medicine, Nashville, Tennessee, United States of America; 2 Osher Center for Integrative Medicine, Vanderbilt University School of Medicine, Nashville, Tennessee, United States of America; 3 Department of Psychiatry, Vanderbilt University School of Medicine, Nashville, Tennessee, United States of America; 4 Institute for Psychiatric Neuroscience, Nashville, Tennessee, United States of America; University of Lisbon, PORTUGAL

## Abstract

The cytokines IL-1α and IL-1β exert powerful pro-inflammatory actions throughout the body, mediated primarily by the intracellular signaling capacity of the interleukin-1 receptor (IL-1R1). Although *Il1r1* knockout mice have been informative with respect to a requirement for IL-1R1 signaling in inflammatory events, the constitutive nature of gene elimination has limited their utility in the assessment of temporal and spatial patterns of cytokine action. To pursue such questions, we have generated C57Bl/6J mice containing a floxed *Il1r1* gene (*Il1r1*^*loxP/loxP*^), with loxP sites positioned to flank exons 3 and 4 and thereby the ability to spatially and temporally eliminate *Il1r1* expression and signaling. We found that *Il1r1*^*loxP/loxP*^ mice breed normally and exhibit no gross physical or behavioral phenotypes. Moreover, *Il1r1*^*loxP/loxP*^ mice exhibit normal IL-1R1 receptor expression in brain and spleen, as well as normal IL-1R1-dependent increases in serum IL-6 following IL-1α injections. Breeding of *Il1r1*^*loxP/loxP*^ mice to animals expressing a cytomegalovirus (CMV)-driven Cre recombinase afforded efficient excision at the *Il1r1* locus. The *Il1r1*^*loxP/loxP*^ line should be a valuable tool for the assessment of contributions made by IL-1R1 signaling in diverse cell types across development.

## Introduction

Interleukin-1 (IL-1) is one of several pro-inflammatory cytokines involved in mediating physiologic responses to injury, infection and inflammation [1, 2]. There are two distinct subtypes of biologically active IL-1, IL-1α and IL-1β, both of which share similar signaling and biologic function, though these peptides only share approximately 25% identity at the amino acid level [3–5]. The induction of IL-1 expression has widespread pro-inflammatory effects including the induction of hyperthermia, pain, a decrease in systolic blood pressure, leukocytosis and sickness behavior [6, 7]. Although believed to be beneficial in normal physiologic responses to injury or infection, abnormal, prolonged induction of IL-1 is associated with several diseases that have a distinct inflammatory component such as rheumatoid arthritis, autoimmune disorders, heart disease and gout [7–10].

The IL-1 receptors (IL-1R) that mediate IL-1 signaling are of three distinct subtypes, denoted IL-1R type 1 (IL-1R1), IL-1R type 2 (IL-1R2) and IL-1R type 3 (IL-1R3) [11–14]. The IL-1R1 is a ubiquitously expressed 576 amino acid, 80 kDa receptor [2, 11], that contains three domains, all of which are involved in ligand binding [15]. There are four known promoters that regulate the expression of murine IL-1R1 isoforms (P1-P4), with the use of P3 producing a receptor with 43 additional amino acids at the N-terminus, compared to P1 and P2 promoter-driven receptors [14, 16]. These promoters are believed to act in a cell and tissue specific manner, one of which (P4) results in a truncated IL-1R primarily located within the CNS, denoted IL-1R3 [14, 16]. IL-1R1 is the generally accepted receptor through which both IL-1α and IL-1β independently initiate immune-driven intracellular signaling through conserved cytoplasmic regions denoted as Toll- and IL-1R-like (TIR) domains [2]. Activation of IL-1R1 by IL-1 results in the recruitment of the IL-1R accessory protein (IL-1RAcP) that subsequently recruits the intracellular signaling proteins myeloid differentiation primary response gene 88 (MyD88) and interleukin-1 receptor-activated protein kinase 4 (IRAK4). This complex containing both MyD88 and IRAK4 is required for IL-1R1-mediated signaling [2, 17]. IL-1R signaling through this complex ultimately results in the activation of p38 MAPK, c-Jun N-terminal kinase (JNK) and NF-κB linked pathways, resulting in a rapid induction of gene expression, including the activation of a positive feedback loop whereby the expression of IL-1α and IL-1β is increased [2]. These processes are believed to be under the strict control of distinct regulatory mechanisms including the presence of a third biologically inactive subtype of IL-1, termed IL-1R antagonist (IL-1RA) [2, 18, 19]. IL-1RA binds to IL-1R1 with similar affinity to its biologically active counterparts, though binding fails to initiate downstream, intracellular signaling [20]. The second subtype of IL-1R, IL-1R2 is a key regulator of signaling involving IL-1. IL-1R2 proteins have a truncated intracellular domain that consists of only 29 amino acids, and are therefore unable to initiate the intracellular signaling cascades discussed above in response to IL-1 binding [20, 21]. The expression of IL-1R2 appears to be relegated specifically to cells of the immune system such as neutrophils, microglia, T regulatory cells (Treg’s) and monocytes, conferring specificity in quenching the inflammatory actions of IL-1 in immune-mediated functions, leaving unopposed the more widely expressed IL-1R1 [22–25]. Lastly, IL-1R3 has been shown to be expressed primarily in neural tissue where it utilizes a second subtype of IL-1RAcP, dubbed IL-1RAcPb, to rapidly activate intracellular protein kinase B (Akt), whereby it modulates voltage-gated potassium currents [14].

The broad expression of IL-1R1 in assorted cell types [6, 26] is believed to underlie the diverse effects that accompany production and secretion of IL-1 peptides. Two lines of constitutive *Il1r1* knockout mice (*Il1r1*^-/-^) have been generated and have proven useful in defining a requirement for IL-1R1 in many effects elicited by IL-1 peptides [27, 28]. The ubiquitous elimination of IL-1R1 expression is problematic however, when seeking to establish the temporal and/or spatial requirements for IL-1R1 signaling. Here, we describe the generation and characterization of mice with a floxed allele of the *Il1r1* gene that is suitable for the conditional elimination of *Il1r1* expression, allowing for the spatial and temporal manipulation of IL-1R signaling.

## Methods

### Generation of *Il1r1*^*loxP/loxP*^ mice

**Fig 1:**
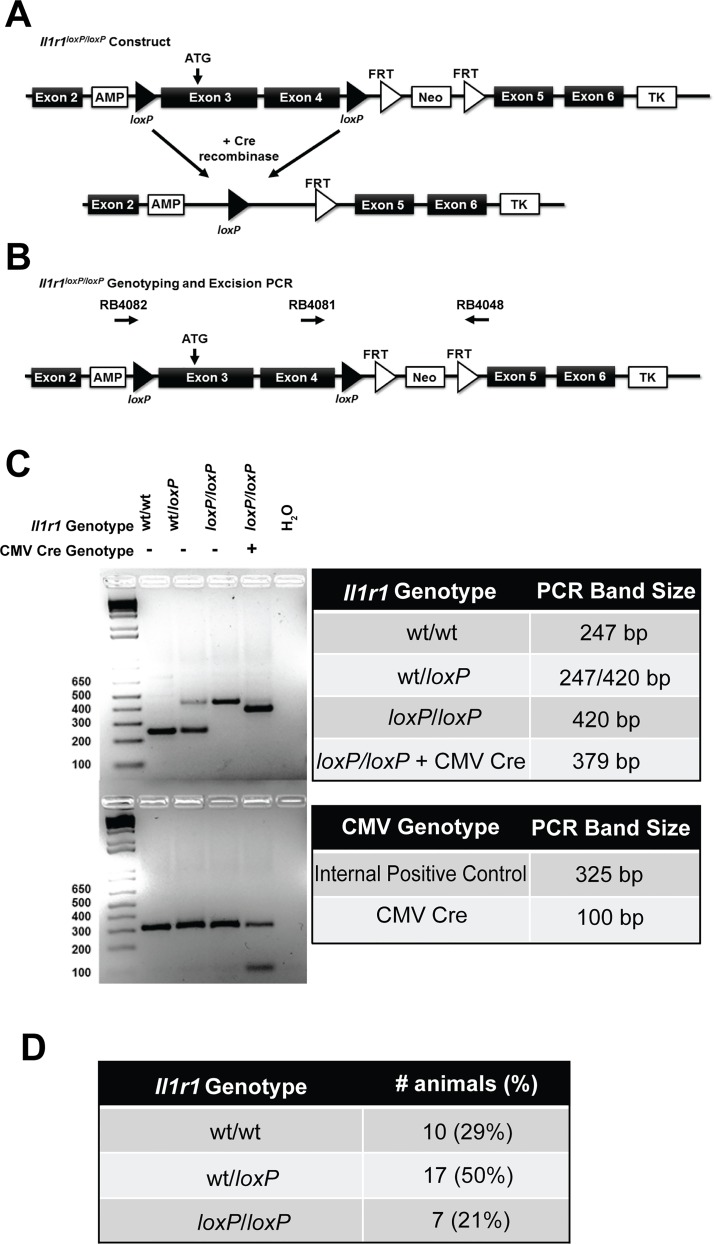
Targeting Construct and Genotyping of *Il1r1*^*loxP/loxP*^ and *Il1r1*^loxP/loxP^:CMV Cre Mice. (A/B) Targeting construct illustrating position of *loxP* sites flanking exons 3 and 4 of the *Il1r1* gene and presence of positive (Neo^r^) and negative (TK) selection cassettes. Excision of exons 3 and 4 results in an elimination of the expression of downstream exons and thereby *Il1r1* function. Diagram also includes locations of sense (RB4082 and RB4081) and antisense (RB4048) oligonucleotide primers utilized for both genotyping and the confirmation of excision of *Il1r1* exons 3 and 4 and subsequent ligation. (C) PCR-based genotyping of *Il1r1*^*loxP/loxP*^ and *Il1r1*^*loxP/loxP*^:CMV Cre mice. The WT locus yields an amplicon of 247 bp, whereas PCR of the floxed allele yields an amplicon of 420 bp. Detection of excised floxed *Il1r1* alleles results from the utilization of primer 1 (RB4082, sense) and primer 3 (RB4048, antisense) to produce an amplicon of 379 bp. (D) Litters born to *Il1r1*^*wt/loxP*^ pairings produce offspring at the normal, expected Mendelian frequency.

All experiments involving animal subjects were conducted as approved by the Vanderbilt Institutional Animal Care and Use Committee. To generate *Il1r1*^*loxP/loxP*^ mice, we utilized a homologous recombination approach, targeting the *Il1r1* gene in TL-1 129S6/SvEvTac embryonic stem cells (ES) in the Vanderbilt Transgenic Mouse/Embryonic Stem Cell Shared Resource (https://labnodes.vanderbilt.edu/community/profile/id/8). The targeting construct (Fig 1A) was generated with 129S6 genomic DNA, and contains *loxP* sites flanking exons 3 and 4, along with an inverted neomycin resistance (Neo^r^) cassette for positive selection of transformants. Exon 3 contains the ATG start site for the functional *Il1r1* gene (Fig 1A) and the successful excision of exons 3 and 4 results in the complete elimination of downstream *Il1r1* exon expression. The (Neo^r^) cassette was flanked by FRT sites to afford Flp-mediated excision. A thymidine kinase (TK) cassette was also included to allow for negative selection. Cassette arms were 4.4 kb for the 5’ arm and 6.3 kb for the 3’ arm. Following electroporation and selection, positively-targeted colonies were identified by Southern blotting, PCR and DNA sequencing. One correctly targeted cell line was selected for microinjection into the blastocoel cavity of 3.5 day old embryos (C57Bl/6J) and subsequently transferred to the uterus of a pseudopregnant female (ICR) mouse. Resulting pups were screened to identify potential founders using PCR, with positive offspring mated to C57Bl/6J animals to achieve germline transmission, followed by subsequent breeding to C57Bl/6J mice that express Flp recombinase (Jackson Labs, Bar Harbor, Maine). Offspring were genotyped to verify successful elimination of Neo^r^ and were then backcrossed for 10 generations on a C57Bl/6J background. We used *Il1r1*^*loxP/loxP*^ mice of either sex at 8–16 wks of age unless otherwise noted, deriving animals homozygous for a floxed *Il1r1* gene from heterozygous breeding pairs to afford littermate wild type controls. Homozygous *Il1r1*^-/-^ breeders (Jackson Labs, B6.129S7-*Il1r1*^tm1Imx^/J, strain number 003245) were used to generate all *Il1r1*^-/-^ animals used in the study, with studies utilizing these mice employing males of 8–16 wks of age. Additionally, *Il1r1*^*loxP/loxP*^ mice generated during the course of these studies have been deposited at Jackson Labs (Bar Harbor, Maine) for dissemination to the greater research community.

### Genotyping of *Il1r1*^*loxP/loxP*^ mice and *Il1r1*^*loxP/loxP*^:CMV Cre mice

Mice were weaned at 21 days of age and a 3 mm tail section was removed for DNA extraction. REDExtract-N-Amp Tissue PCR kits (Sigma-Aldrich, St. Louis, MO) were used for all genotyping procedures following manufacturer’s instructions. To determine *Il1r1* genotype, oligonucleotide primers used for PCR-based genotyping were as follows: 5’-GTGCTAGAACATCCTTTGAGAG-3’ (sense, RB4081) and 5’-GTACCAATGGAGGCCAGAAG-3’ (antisense, RB4048, Fig 1A). All *Il1r1* genotype PCR reactions were performed on a MJ Research PTC-200 Thermal Cycler using the following conditions: initial denaturing step of 94°C for 3 min, followed by 35 cycles of 94°C for 30 sec, 57°C for 30 sec, 72°C for 30 sec, followed by a final extension step of 72°C for 5 min. Sequences for oligonucleotide primers to determine CMV Cre genotyping were obtained from Jackson Labs and were as follows: 5’-GCGGTCTGGCAGTAAAAACTATC-3’ (sense, RB4844) and 5’-GTGAAACAGCATTGCTGTCACTT-3’ (antisense, RB4845). Oligonucleotide primers for the internal positive control were as follows: 5’-CTAGGCCACAGAATTGAAAGATCT-3’ (sense, RB4846) and 5’-GTAGGTGGAAATTCTAGCATCATCC-3’ (antisense RB4847). CMV Cre genotyping PCR reactions were performed on a MJ Research PTC-200 Thermal Cycler using the following conditions: initial denaturing step of 94°C for 3 min, followed by 35 cycles of 94°C for 30 sec, 52°C for 1 min, 72°C for 1 min, followed by a final extension step of 72°C for 2 min. All samples were then resolved on a 2% agarose gel to determine genotype, with wild type alleles producing a 247 bp amplicon and floxed alleles producing a 420 bp amplicon.

### Excision of floxed *Il1r1* allele

To ascertain whether the floxed *Il1r1* locus supports efficient Cre recombinase mediated excision, *Il1r1*^*loxP/loxP*^ dams were bred to a C57Bl/6J sire expressing Cre recombinase via a cytomegalovirus (CMV) promoter (B6.C-Tg(CMV-Cre)1Cgn/J, Jackson Labs, Bar Harbor, Maine). DNA from the resulting pups *Il1r1*^*wt/loxP*^:CMV-cre or *Il1r1*^*wt/loxP*^, was evaluated for IL-1R1 excision using the protocol described above for genotyping of wild type and *Il1r1*^*loxP/loxP*^ animals. DNA was also assessed for the presence of CMV-cre by PCR according to vendor’s recommendations (Jackson Labs, Bar Harbor, Maine). Sequences for oligonucleotide primers to determine *Il1r1*^*loxP/loxP*^:CMV Cre genotyping were the same as those described above (RB4048 and RB4081 for *Il1r1* genotype and RB4844 and RB4845 for CMV Cre genotype). In order to provide evidence for the successful excision and ligation of floxed alleles by CMV cre, *Il1r1*^*loxP/loxP*^:CMV cre mice were obtained from *Il1r1*^*loxP/loxP*^ x *Il1r1*^*loxP/loxP*^:CMV Cre breedings. Genotyping was conducted on these mice using the addition of a third primer upstream of the floxed alleles, within the context of the standard *Il1r1*^*loxP/loxP*^ genotyping protocol described above, to obtain a 379 bp PCR product as a result of excision of floxed *Il1r1* alleles (Fig 1A and 1B). Sequence of this third oligonucleotide primer was as follows: RB4082, 5’-CTTGTGTCCTATGGGTGTCC-3’ (sense).

### Behavioral analyses

To determine whether insertion of *loxP* sites resulted in any gross physiologic or behavioral effects, we utilized a modified form of an Irwin behavior screen [29]. Our screen consisted of assessments of body temperature, spontaneous behavior in a novel environment, righting reflexes, touch escape, toe and tail pinch, weight, visual function and negative geotaxis, conducted as previously described [30]. Briefly, the general physical condition and characteristics of each respective mouse was assessed for the presence of whiskers, quality of fur, general coat condition, bald patches, and/or piloerection. Limb tone and body tone were also assessed and all observations occurred in a clean cage containing corncob bedding. Body temperature was assessed using a rectal thermometer at 10 wks of age (Physitemp Instruments Inc., Clifton, NJ). Weight was assessed in animals beginning at weaning (21 days of age) and tracked until adulthood at 8 weeks of age. The wire hang test was conducted as previously described [30]. Briefly, mice established a grip on a wire screen that was then inverted 60 cm over a clean plastic cage containing fresh corncob bedding. The latency to fall was recorded up to 60 sec, at which point the mice were removed from the apparatus and returned to the home cage. Motor coordination was assessed using an accelerating Rotarod apparatus (Ugo Basile, Comerio VA, Italy). Mice were placed on the rotating cylinder (3 cm in diameter) with the rotational speed of the cylinder increasing from 5 to 40 rpm over the 5-min testing period. The latency at which each respective mouse fell off of the rotating cylinder was assessed. Each mouse was given a single trial on the Rotarod before performance was scored. Locomotor activity was assessed using open field locomotor chambers (11.0 x 11.0 inches, MedAssociates OFA-510, St. Albans, VT) containing 16 infrared photobeam detectors for a total of 1 hr and analyzed with associated software. All behavioral analyses were performed in the Vanderbilt Mouse Neurobehavior Core (https://medschool.vanderbilt.edu/mouse-core/).

### Quantitative real-time PCR analysis of *Il1r1* mRNA expression

Quantitative real-time PCR (qRT-PCR) was utilized to determine *Il1r1* mRNA expression in wild type and *Il1r1*^*loxP/loxP*^ animals. Midbrain and spleen samples were collected from both male and female mice that were sacrificed by rapid decapitation, with tissues flash frozen using liquid nitrogen, prior to storage at -80°C. RNA isolations were conducted from tissue samples using Trizol reagent (Invitrogen, Grand Island, NY) according to manufacturer’s instructions. The total RNA concentration for each sample was quantified by spectrophotometry using a NanoDrop (Thermo Scientific, Wilmington, DE), with the purity of samples checked to confirm that the 260/280 ratio was in the range of 1.8–2.1. Quantitative real-time PCR was conducted using a KAPA SYBR-FAST qRT-PCR One-Step Kit according to manufacturer’s instructions (KAPA Biosystems, Wilmington, MA). Thermocycling conditions were as follows: 42°C for 5 min for cDNA synthesis, 95°C for 5 min for denaturation, followed by 40 cycles of 95°C for 3 sec, followed by annealing and extension at 60°C for 30 sec. Melting curves were obtained for all samples over a period of 15 sec at 95°C, 15 sec at 55°C, followed by a final period of 15 sec at 95°C. All experiments were conducted using an Eco qRT-PCR machine (Illumina, San Diego, CA). Oligonucleotide primers were obtained from Sigma-Aldrich (St. Louis, MO) and were as follows: *Il1r1* (Forward: RB4791, 5’-GCACGCCCAGGAGAATATGA-3’, Reverse: RB4792 5’-AGAGGACACTTGCGAATATCAA-3’). Primers were designed based upon input template NM_001123382.1 and have been validated herein utilizing *Il1r1*^-/-^ samples as controls. Data were normalized to *Gapdh* levels measured in parallel using primers as follows (Forward: RB4748 5’-GGAAGGGCTCATGACCACAG-3’, Reverse: RB4749 5’-TCACGCCACAGCTTTCCAG-3’). Sample mRNA levels from qRT-PCR assays were calculated using the ΔΔC_t_ method [31] and normalized compared to either wild type littermate or *Il1r1*^*-/-*^ counterparts. Threshold levels were determined automatically (Illumina EcoStudy 5.0 software, San Diego, CA) and the threshold cycle (C_t_ value) of each gene was then normalized to *Gapdh* expression.

### Western blotting

Male, littermate wild type and *Il1r1*^*loxP/loxP*^ mice obtained from heterozygous breeding pairs and *Il1r1*^-/-^ mice obtained from homozygous breeding pairs were sacrificed by rapid decapitation and whole spleens resected. Freshly dissected spleens were homogenized in a glass Teflon homogenizer with 3 mL of ice-cold 50 mM Tris, pH 7.4, 1 mM EDTA, 10% sucrose and protease inhibitors (P8340, 1:100; Sigma, St. Louis, MO). Homogenates were centrifuged at 10,000 *X g* for 10 min at 4°C. The resulting membrane pellet was washed in 3–5 mL of ice-cold 50 mM Tris pH 7.4, 1 mM EDTA buffer containing protease inhibitors. Membrane samples were subsequently centrifuged at 10,000 X g for 10 minutes. Pellets containing membrane fraction were solubilized in RIPA buffer (50 mM Tris, pH 7.4, 150 mM NaCl, 1 mM EDTA, 1% TRITON X-100, 1% sodium deoxycholate, 0.1% SDS) for 1 h at 4°C. Protein lysates were centrifuged at 4°C for 10 min at 15,000 *x g* to remove insoluble material. Protein concentrations were determined using the BCA method (ThermoFisher, Waltham, MA) and 40 μg of total protein was separated by 10% SDS-PAGE, transferred to PVDF (Bio-rad, Hercules, CA). Membranes were blocked using 5% dry milk in PBS-T for 2 hr at room temp. Membranes were incubated with primary IL-1R1 antibody (1:500; Santa Cruz IL-1R1 M20; SC689) overnight at 4°C and washed 3 x 10 min with PBS-T. Secondary antibody was utilized at a dilution of 1:2000 (Jackson Immunoresearch; goat anti-rabbit-HRP). β-Actin was detected using a 1:50,000 dilution of β-actin-HRP antibody (Sigma, A3854). Blots were once again washed 3 x 10 min with PBS-T prior to image capture. Immunoreactive bands were identified by chemiluminescence and imaged with ImageQuantTM LAS400 (GE Healthcare Life Sciences, Pittsburg, PA). IL-1R1 expression was normalized to β-actin and analysis was conducted utilizing Image J software.

### *In vivo* IL-1R1 functional characterization

To assess the impact of *loxP* site insertions on the functional capacity of expressed IL-1R1s, we evaluated the ability of recombinant IL-1α to increase serum IL-6 expression *in vivo*, as described by Glaccum et al. [27]. Briefly, male *Il1r1*^*loxP/loxP*^ mice and their wild type littermates were injected i.p. with either vehicle (saline, 0.1 mL total volume) or 1 μg recombinant IL-1α (0.1 mL/animal of a 10 μg/1 mL solution) (GenScript Piscataway, NJ). Two hrs post-injection, mice were sacrificed by rapid decapitation, trunk blood was collected and allowed to sit for 30 min at room temp. To determine whether excision of *Il1r1* alleles by CMV Cre expression results in an elimination of IL-1R1 function, a similar breeding strategy as that described above was utilized to obtain *Il1r1*^*loxP/loxP*^:CMV Cre and *Il1r1*^*loxP/loxP*^ mice. These mice, in conjunction with *Il1r1*^-/-^ animals as negative controls, were treated with IL-1α (1μg) as those described above and two hrs post-injection samples were collected. Samples were then centrifuged at 1500 *x g* for 10 min prior to being stored at -80°C, IL-6 levels were determined on thawed serum samples by ELISA (Mouse Ready-SET-Go IL-6 ELISA, eBioscience, San Diego, California) according to the manufacturer’s instructions with product quantified using a POLARStar Omega microplate spectrophotometer (BMG Labtech, Ortenberg, Germany).

### Statistical analyses

Statistical analyses were conducted using Student’s unpaired t tests for Irwin behavioral screen, qRT-PCR data and average velocity obtained from locomotor activity studies. Distance traveled in open field locomotor studies and data obtained from the *in vivo* IL-1R1 signaling assays were analyzed using either one-way or two-way analysis of variance (ANOVA), followed by post hoc Bonferroni’s or Tukey’s multiple comparison tests, respectively. Data was analyzed and graphed using GraphPad Prism 5 (GraphPad Software, La Jolla, CA). *P* values < 0.05 were considered indicative of a statistically significant effect.

## Results

### Generation of *Il1r1*^*loxP/loxP*^ mice

**Fig 2:**
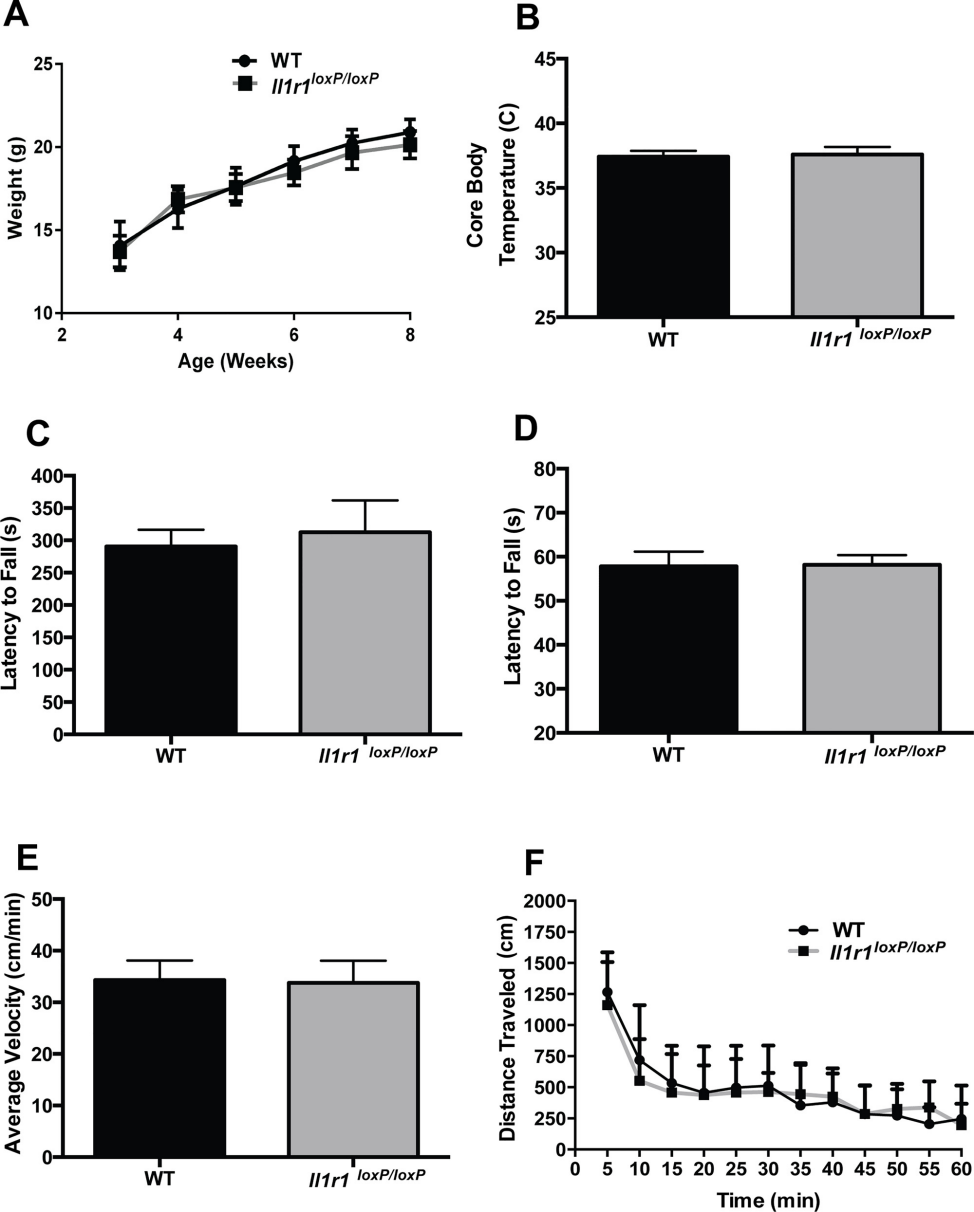
Basic Phenotyping of *Il1r1*^*loxP/loxP*^ Mice. Adult *Il1r1*^*loxP/loxP*^ mice display normal (A) development as indicated by weight gain until adulthood as compared to wild type littermate counterparts (two-way repeated measures ANOVA, followed by post hoc Tukey’s multiple comparison tests, N = 7-10/group, *P >* 0.05) (B), body temperature (C), Rotarod performance and (D) hang time in the inverted screen test (unpaired Student’s t test, N = 12/group, *P* > 0.05). Additionally, *Il1r1*^*loxP/loxP*^ mice exhibit comparable rates of locomotor activity to WT littermates when evaluated as (E) average velocity (Student’s unpaired t test, *P* > 0.05) or (F) distance traveled, two-way ANOVA, N = 6/group, genoptype effect, P > 0.05).

As shown in Fig 1C, we successfully achieved *loxP* site insertion at the *Il1r1* locus, findings also confirmed by direct DNA sequencing (data not shown). Breeding of C57Bl/6J congenic heterozygous animals (*Il1r1*^wt/loxP^) resulted in expected litter sizes which followed Mendelian expectancies (Fig 1D). Offspring of these breedings displayed no overt growth or viability issues, as indicated by a normal body weight trajectory as compared to their wild type littermate counterparts (Fig 2A). These findings were expected as constitutive *Il1r1*^-/-^ mice breed normally, are born in normal litter sizes at the expected Mendelian frequency and display no overt phenotypes [27, 28]. We also observed no differences in body temperature (Fig 2B), in duration of mobility in the accelerating Rotarod test (Fig 2C), or in latency to fall from an inverted screen (Fig 2D). Other aspects of the Irwin battery [29, 30], such as negative geotaxis, reflexes, spontaneous behaviors or visual function were normal. Additionally, we detected no significant differences in locomotor activity in the open field (average velocity or total distance traveled) comparing *Il1r1*^*loxP/loxP*^ mice with their wild type littermates (Fig 2E and 2F). Together, these studies suggest minimal to no overt physiological or behavioral effects of the *Il1r1*^*loxP/loxP*^ genotype.

### Assessment of *Il1r1* mRNA and protein expression in *Il1r1*^*loxP/loxP*^ mice

**Fig 3:**
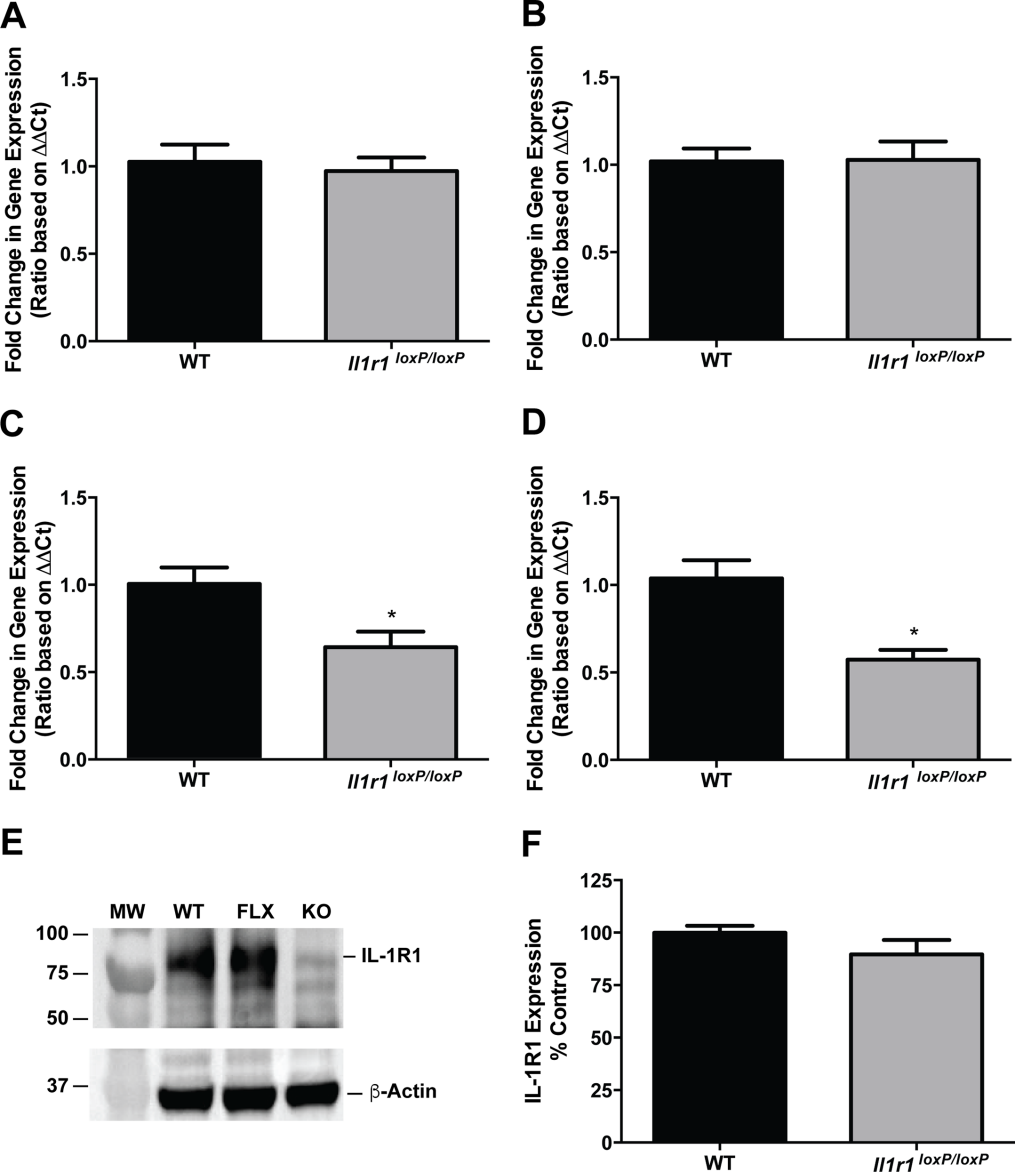
*Il1r1*^*loxP/loxP*^ Mice Exhibit Normal *Il1r1* mRNA Expression in the CNS, but Lower Splenic *Il1r1* mRNA Levels without a Concomitant Decrease in IL-1R1 Protein Expression. qRT-PCR was utilized to determine *Il1r1* mRNA levels in midbrain and spleen samples from male and female *Il1r1*^*loxP/loxP*^ and wild type littermates. Midbrain samples from both male (A) and female (B) *Il1r1*^*loxP/loxP*^ mice exhibit comparable levels of *Il1r1* mRNA expression as wild type littermates (Student’s unpaired t test, N = 14/group, *P* > 0.05). In contrast, both male (C) and female (D) *Il1r1*^*loxP/loxP*^ mice exhibit significantly reduced levels of splenic *Il1r1* mRNA expression as compared to wild type littermates (Student’s unpaired t test, N = 6-8/group, * = *P* < 0.05). Western blot analysis however, revealed *Il1r1*^*loxP/loxP*^ spleen samples to express normal levels of IL-1R1 protein (E) as compared to their wild type littermates (quantified in F, Student’s unpaired t test, N = 6/group, *P* > 0.05).

To determine whether the insertion of *loxP* sites flanking exons 3 and 4 of the *Il1r1* gene resulted in any disruptions in the expression of receptor mRNA or protein, we pursued qRT-PCR and western blot analyses, respectively. The *Il1r1* gene is broadly expressed, including the CNS, where IL-1R1 receptor signaling has been implicated in several neurodegenerative disorders such as Alzheimer’s, Parkinson’s and multiple sclerosis [26, 32, 33]. Our own work has implicated an IL-1R1/p38 MAPK signaling pathway in the regulation of serotonin transporters that are expressed by midbrain raphe neurons [34, 35]. In midbrain extracts, we observed no effect of *loxP* site insertion on *Il1r1* mRNA expression in either male (Fig 3A) or female (Fig 3B) mice. Surprisingly, spleen samples from these same mice displayed 40–50% reductions in *Il1r1* mRNA in both male (Fig 3C) and female (Fig 3D) *Il1r1*^*loxP/loxP*^ mice, relative to their wild type littermates. As mRNA levels do not always correspond with levels of expressed protein, we complemented our qRT-PCR studies with western blot analyses. As shown in Fig 3E (quantified in Fig 3F), these studies revealed no significant differences in splenic IL-1R1 protein expression evident between wild type and *Il1r1*^*loxP/loxP*^ littermates.

### Confirmation of excision of floxed *Il1r1* alleles

**Fig 4:**
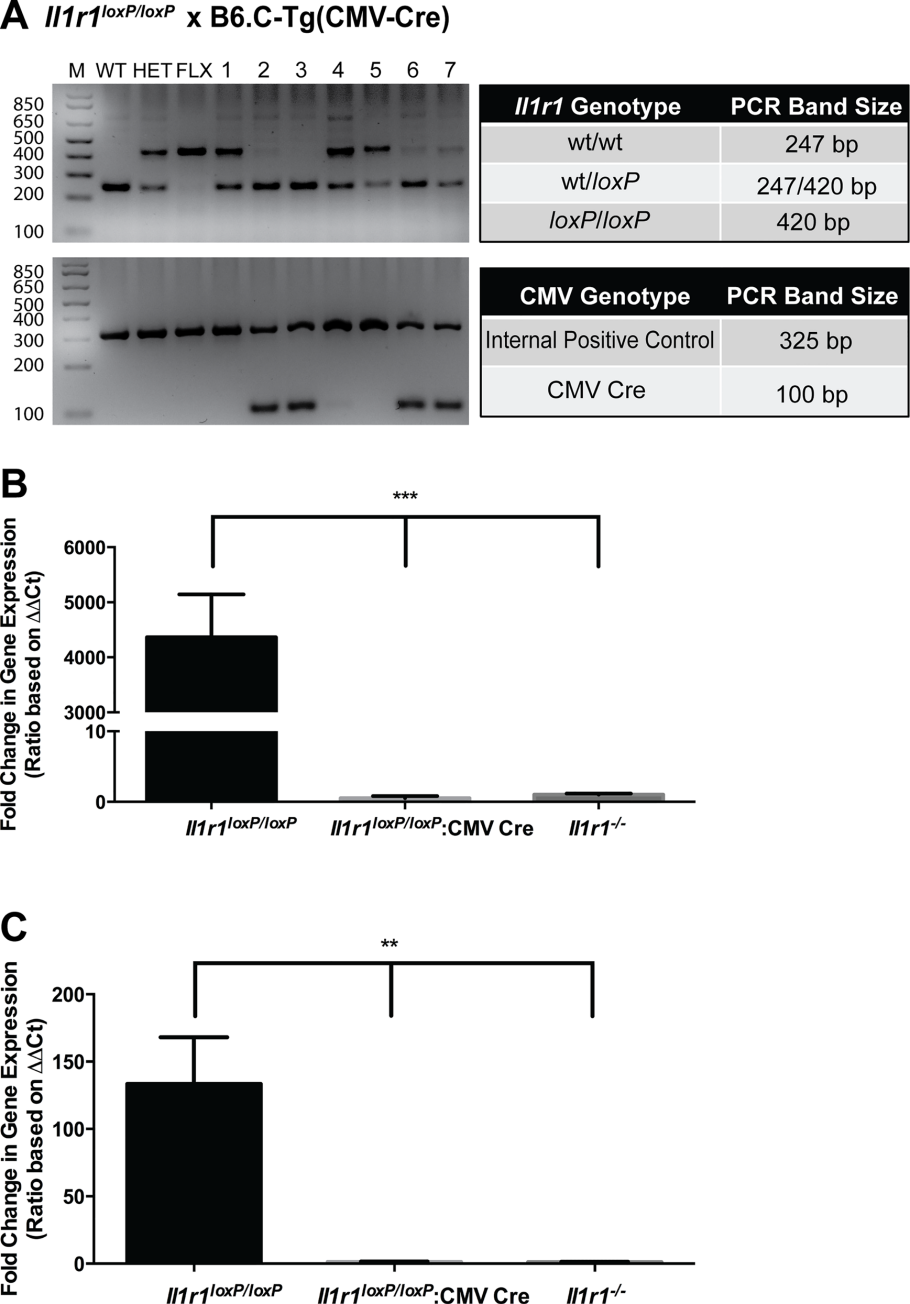
Excision of Floxed *Il1r1* Alleles by Cre Recombinase and Elimination of *Il1r1* mRNA Expression in *Il1r1*^*loxP/loxP*^:CMV Cre Mice. (A) *Il1r1* genotyping of mice born to a cross of *Il1r1*^*loxP/loxP*^ and CMV-Cre mice revealed offspring to be of two distinct genotypes, *Il1r1*^wt/loxP^:CMV-Cre or *Il1r1*^wt/loxP^. All pups expressing Cre recombinase displayed excision of floxed *Il1r1* alleles (lanes animals 2, 3, 6 and 7). M = Molecular weight marker, WT = wildtype control sample, HET = heterozygous (*Il1r1*^wt/loxP^) control sample (lacking Cre), FLX = homozygous (*Il1r1*^*loxP/loxP*^) control sample (lacking Cre). Actual band sizes–unexcised floxed allele = 420 base pairs, WT allele = 247 base pairs. (B) Global expression of Cre recombinase driven through the CMV promoter results in the elimination of splenic *Il1r1* mRNA expression in *Il1r1*^*loxP/loxP*^:CMV Cre mice. (One way ANOVA, *P* < 0.0001, post hoc Tukey’s multiple comparison tests, *** = *P* < 0.0001; N = 5-7/group) (C) Global expression of Cre recombinase driven through the CMV promoter results in the elimination of *Il1r1* mRNA expression in the midbrain of *Il1r1*^*loxP/loxP*^:CMV Cre mice. (One way ANOVA, P < 0.01, post hoc Tukey’s multiple comparison tests, * = *P* < 0.01; N = 5-7/group).

To determine whether floxed *Il1r1* alleles support excision by Cre recombinase, we crossed *Il1r1*^*loxP/loxP*^ homozygous mice to mice expressing Cre recombinase under the control of a CMV promotor (B6.C-Tg(CMV-Cre)1Cgn/J, strain number 006054, Jackson Labs, Bar Harbor, Maine). The CMV promoter results in excision of floxed alleles in all tissues, including germ cells. Genotyping of pups from the aforementioned cross (Fig 4A) revealed relatively complete excision of the floxed *il-1r1* allele in animals that expressed Cre recombinase (Samples 2, 3, 6 and 7, Fig 4A). These results, in conjunction with excision results noted above (Fig 1B), provide clear evidence that floxed *Il1r1* alleles are able to be excised efficiently at the genetic (DNA) level.

We next sought to determine whether floxed *Il1r1* alleles afforded complete excision of *Il1r1* expression at the mRNA level in *Il1r1*^*loxP/loxP*^:CMV Cre mice. To determine whether transcription of the *Il1r1* gene, we conducted qRT-PCR as described above. This analysis revealed that in both the spleen (Fig 4B) and midbrain (Fig 4C), CMV Cre expression in *Il1r1*^*loxP/loxP*^:CMV Cre mice resulted in an elimination of *Il1r1* mRNA consistent with the constitutive elimination of *Il1r1* mRNA expression in *Il1r1*^*-/-*^ mice.

### Characterization of *in vivo* IL-1R1 function in *Il1r1*^*loxP/loxP*^ mice

**Fig 5:**
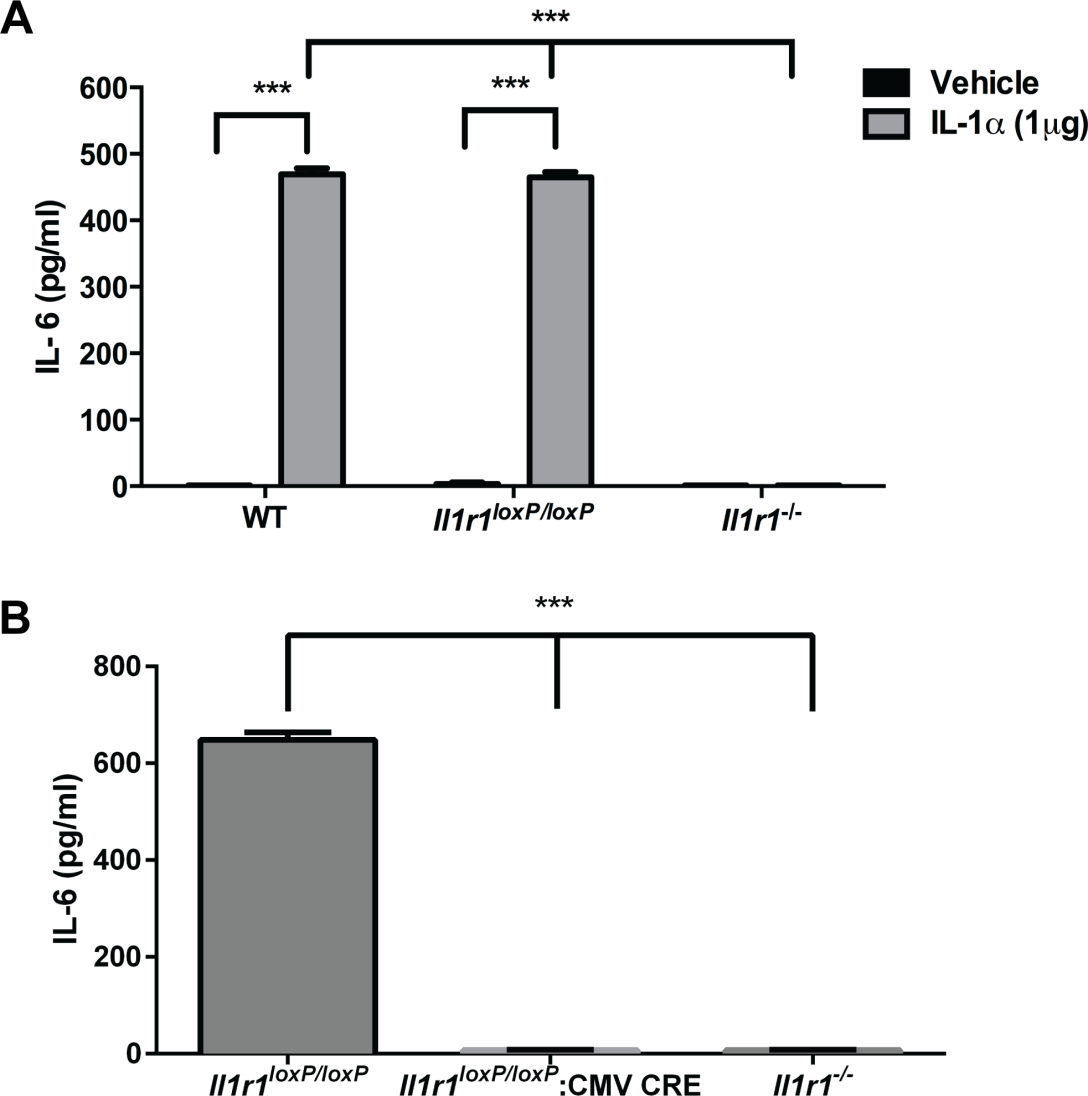
*Il1r1*^*loxP/loxP*^ Mice Exhibit Normal IL-1R1-Mediated Increases in Serum IL-6 Following IL-1α Injections. (A) WT and *Il1r1*^*loxP/loxP*^ animals exhibit significant and comparable serum IL-6 elevations 2 hrs after IL-1α injections (1 μg, i.p.), as compared to vehicle treated controls (two-way ANOVA, *P* < 0.0001 for treatment, genotype and their interaction, followed by post hoc Tukey’s multiple comparison tests, *** = *P* < 0.0001). IL-1α induced IL-6 elevations were found to be IL-1R1 dependent, as revealed by a lack of response in *Il1r1*^-/-^ animals. N = 4-6/group for all assays. (B) Excision of floxed *Il1r1* alleles by global Cre recombinase expression in *Il1r1*^*loxP/loxP*^ mice attenuates the ability of IL-1α treatment to increase serum IL-6 expression. No increases in IL-6 expression were found in IL-1α treated *Il1r1*^*loxP/loxP*^:CMV Cre or *Il1r1*^*-/-*^ mice (one way ANOVA, *P* < 0.0001, followed by post hoc Tukey’s multiple comparison tests, *** = *P* < 0.0001) N = 5-7/group for all assays.

Following the assessment of IL-1R1 expression levels in *Il1r1*^*loxP/loxP*^ mice, we sought to confirm that these animals exhibit physiologically normal responses to the activation of IL-1R1. To pursue this goal, we injected male, *Il1r1*^*loxP/loxP*^ mice, their wild type littermates, and constitutive *Il1r1*^-/-^ animals with either IL-1α or vehicle and determined serum IL-6 levels 2 hrs post-treatment. IL-1α-induced increases in serum IL-6 are dependent upon the presence of functional IL-1R1 as previously demonstrated using constitutive *Il1r1*^-/-^ mice [27]. As shown in Fig 5, vehicle treated animals of all three genotypes display nearly undetectable levels of serum IL-6. Treatment of wild type animals with IL-1α resulted in a large increase in circulating IL-6 levels in the serum 2 hrs post-treatment, an effect that was found to be dependent upon intact IL-1R1 function, as treatment of constitutive *Il1r1*^-/-^ animals failed to elicit detectable IL-6 elevations. *Il1r1*^*loxP/loxP*^ mice were also found to exhibit significant IL-1α-induced increases in serum IL-6 compared to vehicle, an increase equivalent to that seen with wild type littermates. These data corroborate studies of IL-1R1 protein expression and provide evidence of sufficient IL-1R1 expression to maintain a normal *in vivo* IL-1R signaling capacity.

Once we confirmed that *Il1r1*^*loxP/loxP*^ mice exhibit normal physiologic IL-1R1 function, we sought to determine whether excision of IL-1R1 in *Il1r1*^*lox/loxP*^:CMV Cre mice results in the attenuation of IL-1α-induced increases in serum IL-6 expression. As shown in Fig 5B, treatment of *Il1r1*^*loxP/loxP*^ mice with IL-1α (1μg) resulted in a substantial increase in serum IL-6 expression. This effect is attenuated by the global elimination of IL-1R1 in *Il1r1*^*loxP/loxP*^ mice through the expression of Cre recombinase driven by the CMV promoter. Additionally, similar to results described above, no increases in IL-6 elicited by IL-1α were found in constitutive *Il1r1*^-/-^ controls (Fig 5B).

## Discussion

Studies using the constitutive elimination of the mouse *Il1r1* gene have provided important evidence of a requirement for IL-1R-mediated signaling in multiple phenomena, including pancreatic islet function in diabetes [36], atherosclerosis [37], splenocyte responses to glucocorticoid treatment [38] and alterations in transporter function within the CNS [35]. Although a powerful tool, mice with constitutive elimination of IL-1R expression preclude evaluation of the temporal or spatial contributions of IL-1R signaling.

Similar to constitutive *Il1r1*^*-/-*^ mice, the *Il1r1*^*loxP/loxP*^ mice we described herein are viable, breed normally and show no overt changes in gross morphological or behavioral phenotypes. *LoxP* site insertion appears not to impact levels of *Il1r1* mRNA within the midbrain, and so we were surprised to detect a significant reduction of *Il1r1* mRNA expression in the spleens of *Il1r1*^*loxP/loxP*^ mice. These effects, however, did not translate into alterations in splenic IL-1R1 protein levels, nor were these animals impaired in their ability to translate IL-1α injections into IL-1R1-dependent increases in serum IL-6 elevations. These results are therefore likely to be indicative of excess receptor *Il1r1* mRNA produced relative to the level of protein needed to achieve normal IL-1 signaling. The basis for the observed reduction is unclear but may reflect a cell-specificity of intronic sequences in efficient mRNA splicing. Even with a modest reduction in mRNA levels, it is likely that proper immune function would be essentially intact as constitutive *Il1r1*^-/-^ mice exhibit no alterations in either lymphoid or hematopoietic cell numbers or function [27, 28]. *Il1r1*^-/-^ mice also exhibit no known deficits in IL-1R1-independent immune system function [27, 28]. Additionally, splenic B cells from *Il1r1*^-/-^ mice exhibit normal proliferation in response to cytokines such as IL-4 [27]. Moreover, a relatively small number of IL-1R1s are found on most IL-1 responsive cells or are needed to support normal levels of IL-1 signaling [39, 40]. Of course, vigilance for undesired consequences of genetic manipulations is always advisable when using any genetically modified model. Thus we recommend where possible the use of littermate *Il1r1*^*loxP/loxP*^ mice that lack Cre expression as the reference population against which the effects of Cre-mediated receptor excision are assessed.

The studies reported here demonstrate efficient excision of the *Il1r1*^*loxP/loxP*^ gene in animals bred to co-express Cre recombinase, supporting the feasibility of moving forward with conditional elimination strategies using tissue-specific or developmentally regulated Cre lines. In addition to the obvious utility in elucidating the role of IL-1R1 in specific cells of the immune system, mice described here will be useful in delineating the role of IL-1R1 in various aspects of neuronal function within the CNS. The expression of IL-1R1 in multiple regions and cell types of the CNS has been reported [26, 41, 42] raising questions as to specific circuits engaged in translating inflammatory insults into behavioral changes. For example, IL-1R1 signaling has been linked to depressive-like behaviors elicited by inflammatory insults in clinical cohorts and rodent models [35, 43]. Consistent with this, immune system challenges and chronic stress paradigms result in IL-1R1-dependent depressive-like behaviors and deficits in hippocampal neurogenesis in rodents [35, 44]. We have shown that the depressive-like behaviors associated with IL-1R1 activation in mice are correlated with an increase in the activity of serotonin transporters (SERT) [34] and that peripheral inflammation can lead to an elevated clearance of serotonin in the CNS [35]. These effects have been shown to be dependent upon IL-1R1 through the use of constitutive *Il1r1*^-/-^ mice [35] however the specific region or cell type required for these effects has yet to be determined. The *Il1r1*^*loxP/loxP*^ mice should also allow for investigations of critical periods for IL-1R1 contributions to inflammatory insults that either initiate or sustain physiological and behavioral alterations. Immune system responses to inflammation and/or infection, such as increases in the expression levels of various cytokines (including IL-1), are known to vary with age in the periphery and CNS [45, 46]. In aging, microglia, the resident immune cells of the CNS, are known to be hypersensitive to systemic insults, an effect that results in increased IL-1β production [47]. Recently, we reported that lifelong anxiety and depressive behavior seen in wild type mice that have been subjected to maternal separation during the first weeks of life is absent in *Il1r1*^-/-^ mice [48], raising questions, now addressable with the *Il1r1*^*loxP/loxP*^ model, as to whether IL-1R1 signaling mediates these effects at the time of separation or throughout life.

The absence of a mouse line supporting conditional excision of *Il1r1* has led to alternative strategies to investigate the cell-specificity of IL-1R1 signaling. One method involves the use of bone marrow transplants into constitutive IL-1R1^-/-^ mice to establish a requirement of IL-1R1 signaling by bone-marrow derived cells for features of atherosclerosis [49]. Outside of the immune system, Quan et al. have utilized the endothelial cell-specific Tie2 promoter and a Tet-On system in conjunction with doxycycline treatments to eliminate IL-1R1 expression specifically in endothelial cells [50]. More recently, Liu et al. have produced mice that allow for cell-specific restoration of IL-1R1 expression via Cre recombinase expression [51]. Two independently derived lines of constitutive *Il1r1* ^-/-^ mice [27, 28] express a residual, shortened version of *Il1r1* mRNA (IL-1R3) [14]. The IL-1R1 restore mice produced by Liu et al. [51] fail to express this truncated version of the receptor, and can therefore be utilized as a more efficient global, constitutive *Il1r1* ^-/-^ animal [51]. Additionally, these mice provide researchers with the ability to study the consequences of IL-1R1-mediated signaling in singular cell types. The *Il1r1*^*loxP/loxP*^ line described above adds to the toolkit available for genetic manipulation of IL-1R1 and allows researchers the capacity to both restore and eliminate IL-1R1 expression under temporal control and in specific cell types.

In conclusion, we have generated and characterized a novel mouse model expressing a floxed *Il1r1* gene. These mice are a valuable resource to the research community and will aid in further delineating the roles of IL-1R1-mediated signaling in various cell types. Given the comorbidity of immune system dysfunction with multiple disorders affecting human health, the *Il1r1*^*loxP/loxP*^ model should have translational relevance, including the examination of immune system-based therapeutics.
